# BioNetApp: An interactive visual data analysis platform for molecular expressions

**DOI:** 10.1371/journal.pone.0211277

**Published:** 2019-02-22

**Authors:** Ali M. Roumani, Amgad Madkour, Mourad Ouzzani, Thomas McGrew, Esraa Omran, Xiang Zhang

**Affiliations:** 1 Department of Computer Science, Gulf University for Science and Technology, Mishref, Kuwait; 2 Bindley Bioscience Center, Purdue University, West Lafayette, IN, United States of America; 3 Department of Computer Science, Purdue University, West Lafayette, IN, United States of America; 4 Qatar Computing Research Institute, Qatar Foundation, Doha, Qatar; 5 Department of Chemistry, University of Louisville, Louisville, KY, United States of America; Weizmann Institute of Science, ISRAEL

## Abstract

**Motivation:**

Systems biology faces two key challenges when dealing with large amounts of disparate data produced by different experiments: the integration of results across different experiments, and the extraction of meaningful information from the data produced by these experiments. An ongoing challenge is to provide better tools that can mine data patterns that could not have been discovered through simple visualization. Such mining capabilities also need to be coupled with intuitive visualization to portray those findings. We introduce a software toolbox entitled BioNetApp to mine these patterns and visualize them across all experiments.

**Results:**

BioNetApp is an interactive visual data mining software for analyzing high-volume molecular expression data obtained from multiple ‘omics experiments. By integrating visualization, statistical methods, and data mining techniques, BioNetApp can perform interactive correlative and comparative analysis along time-course studies of molecular expression data. Correlation analysis provides several visualization features such as Kamada-Kawai, Fruchterman-Reingold Spring embedding network layouts, in addition to single circle, multiple circle and heatmap layouts, whereas comparative analysis presents expression-data distributions across samples, groups, and time points with boxplot display, outlier detection, and data curve fitting. BioNetApp also provides data clustering based on molecular concentrations using Self Organizing Maps (SOM), K-Means, K-Medoids, and Farthest First algorithms.

**Conclusion:**

BioNetApp has been utilized in a metabolomics study to investigate the metabolite abundance changes in alcohol induced fatty liver, where pair-wise analyses of metabolome concentration revealed correlation networks and interesting patterns in the metabolomics dataset. This study case demonstrates the effectiveness of the BioNetApp software as an interactive visual analysis tool for molecular expression data in systems biology. The BioNetApp software is freely available under GNU GPL license and can be downloaded (including the case-study data and user-manual) at: https://doi.org/10.5281/zenodo.2563129.

## Introduction

Experiments conducted in the omics arena generate a substantial amount of data. This information is the key to analyze biological behavior through a systems level understanding in which groups of component biomolecules and pathways are connected and operate interdependently. Representation of relationships among biomolecules is also an intensive field of research in systems biology [1]. Correlation and comparative analyses as well as clustering are key elements in understanding such relationships across experiments. Correlation analysis measures the strength of any relationship between the variables. This analysis is useful in testing hypotheses about cause-effect relationships between molecules. Comparative analysis compares the results between experiments. Finally, clustering helps understand the structure of the data and detect anomalies.

With the increased need to perform analysis on large datasets, more attention has been directed to provide data visualization capabilities on top of data mining techniques. On one hand, data visualization plays a very important role in systems biology; it allows for describing complex interactions in a visually analyzable form, which helps identify patterns that may prove useful in hypotheses generation. Visualization across numerous samples simultaneously allows for discovering new interesting information, where diverse layout display is one adopted method of feasible visualization. On the other hand, datamining is at the intersection of multiple fields of research including machine learning, statistics, and database systems. It serves two important goals, insight and prediction. Insight comes from the ability to identify patterns within the data. Such patterns could include the anomalies and features that stand out from the rest of the data population. Prediction is being able to generate a model that would generalize to a population. Prediction could be either discrete (known as classification) or continuous (known as regression). Data mining on molecular expression data can assist in discovering patterns and anomalies that could not have been discoverable otherwise.

The coupling of data visualization and data mining has led to the burgeoning of Interactive Visual Data Mining (IVDM) [2] which aims at supporting knowledge discovery coupled with rich interactions and feasible visualization. The IVDM tools are used to extract meaningful patterns from data sets. Data mining techniques embedded within those tools enable patterns discovery across different experiments. Several visualization software packages have been developed for the interactive visualization of molecular networks, such as Mzmine2 [3,4], Cytoscape [5,6], Graphle [7], BiologicalNetworks [8,9], PathSys [10], SphinGOMAP [11], Sungear [12], ProteoLens [13], CFinder [14], and BisoGenet [15].These tools can be used to display various biomolecular correlation and interaction networks, however some provide limited data mining capabilities (see Table 1) or they may lack the full integration of both information presentation through visualization and instant information discovery through analytical techniques. Most tools require scientists to generate the visualization data independently (through some data analysis tool) and then start visualizing the data through their software package. Others require the data to be in a specific file format (or for specific spectrometry separation method as LC or GC but not both) without providing the user with the means to assist in the automated creation of such formats, adding a limitation to what type of data the user can readily utilize. A comparison between BioNetApp, Mzmine2, Graphle, and Biological Networks is shown in Table 1.

**Table 1:** A comparison between BioNetApp, Mzmine2, Graphle, and Biological Networks.

Specs	BioNetApp	Mzmine2	Graphle	Biological Networks
Data type supported	Supports all data types represented in a comma or tab delimited text file (csv).	Requires Thermo MSFileReader library for some data types.	Supports network data as nodes and edges in the form of key/value pairs in tables.	Supports values of biological network datasets.
Statistical Analysis	Interactive visual data mining of molecular correlations, comparative, and clustering analysis across samples, groups, and time points.	Basic methods for statistical analysis of processed data. Development of such methods is not considered high priority, as processed data can be exported to a third-party statistical software.	Search and visualization engine to view functional relationship networks predicted by the bioPIXIE system and a collection of microarray data. Lacks mining techniques to discover interesting patterns.	Visualization and analysis services over PathSys. Queries databases for known and predicted interactions. Relies on its querying system for information analysis and discovery with no mining capabilities.
Visualization	Comparative analysis uses boxplot display, outlier detection, and data curve fitting. Clustering uses SOM, K-Means, K-Medoids, and Farthest First algorithms. Correlation uses Kamada-Kawai, Fruchterman-Reingold Spring network layouts, with single/multiple circle and heat map layouts.	Can visualize raw data with peak picking and identification results, such as chromatogram plot and 2D plot Shows detected peaks, peak areas, and pal component analysis plots.	Displays dense biological networks as network graphs. Targeted for gene interaction networks.	Can build pathways and common targets, find intersections with curated pathways, and view genome-scale integrated networks of protein-protein, protein-DNA and genetic interactions networks.
Observing the results	Produced graphic presentations can be exported as images, and the molecular correlation information as simple flat text format.	Can report the quantification results in table form or using charts.	Allows exploring networks, scaling between different details of visualizations, and saving images and data.	Imported network interactions and components can be annotated and saved, along with related graphs.

The main objective of our research was to employ the IVDM approach to develop a user-friendly software package for omics expression data analyses that combines intuitive and interactive visualization with strong instantaneous data mining capabilities, namely BioNetApp. The BioNetApp software toolbox is designed to be independent of the type of mass spectrometry system used, as it operates on input files containing the molecular expression data and features to be studied. By integrating visualization, statistical methods, and data mining techniques, BioNetApp is able to perform interactive correlative, comparative, and clustering analysis combined with time-course studies of molecular expression data. Users are able to seamlessly navigate through the analysis results and dynamically interact with the system. We demonstrate the application of BioNetApp using a metabolomics dataset generated in a study on mice to investigate the metabolite abundance changes in alcohol induced fatty liver. Such observations can be used to test the hypothesis that alcohol exposure can disturb lipid homeostasis at the white adipose tissue (WAT)-liver axis towards triacylglycerol epitomic deposition in the liver. Using BioNetApp analysis to find a possible mechanistic link, such as regulation changes of fold increase (up-regulation), between adipose fat loss and hepatic fat gain in alcoholic fatty liver, would prove such effects of alcohol exposure.

The dataset being used in this paper is from a lab experiment (detailed in [16])on mice at two months old that were given an initial priming dose of ^2^H_2_O for five weeks (stage one, time point 0 week). The mice were then randomly grouped into two cohorts(stage two, a 4-week of alcohol exposure): the control cohort (control liquid diet), referred to as group ‘D’ in the experiment with its samples prefixed with the letter ‘C’; and the test cohort (alcohol liquid diet), referred to as group ‘DE’ with its samples prefixed with the letter ‘T’. There were 5, 5, 3 and 5, 7, 5 mice at time point 0, 2, 4 weeks for the control cohort and the test cohort, respectively.

Pair-wise analyses of metabolome concentration and the use of BioNetApp revealed relevant correlation networks and interesting patterns in this metabolomics dataset. BioNetApp was developed using the Java language to provide cross-platform compatibility and easy deployment.

## Materials and methods

The BioNetApp software package went through two main development stages. At first, a prototype initial system called “SysNet” [17] was developed using C++ language, which demonstrated how the core functionalities could be integrated into one system. It also showed how it could be used in the process of hypothesis generation for ionomics data. The latest development stage produced the current BioNetApp software package, a platform for visualizing and mining various relationships between molecular expression data. BioNetApp includes significant additions and improvements (Demonstrated in the Results Section) over its predecessor, such as:

- Developed using Java language instead of C++ to provide cross-platform compatibility and ease of deployment
- Project creation and management interface with generic data importing capabilities
- Various detailed and fast-loading visualization algorithms
- A wide set of operations to interactively manipulate the visualized data and analyze time-course studies.
- A comprehensive molecule information panel with all available identifiers
- Diverse data filtering methods for molecules, samples, and groups
- Rich data mining capabilities and clustering analysis

BioNetApp provides the user with a project management interface that helps to seamlessly design projects and experiments with added meta-data, and also import data into existing projects from various platforms, given the data is represented in a comma or tab delimited file. This functionality significantly enhanced the user experience, since other software packages requires the data to be in a specific format without providing methods to assist in creating such a format.

The visualization algorithms of BioNetApp include Kamada-Kawai [18], Random, and Fruchterman-Reingold Spring Layouts [19],in addition to single circle, multiple circle and heatmap layouts. BioNetApp also provides a set of operations to interactively manipulate the visualized data, such as selecting and hiding highlighted or orphan nodes, hiding outliers, split-screen for comparing sample groups, and displaying multiple circles for up/down regulation expression data. The addition of an integrated information panel provides detailed information display, including molecule/correlation values, display conditions, and graphs for topological information, node-degree distribution, correlation distribution, and neighborhood connectivity. The enhanced data mining capabilities include the use of Self Organizing Maps (SOM) [20] and K-means [21] to perform data clustering, which aids in discovering interesting data patterns, structures, and assess the level of similarity between molecular expression data. BioNetApp is also capable of analyzing time-course studies by introducing the time feature as one of the means to categorize the data and visualize it across time projections, with the ability to create groups based on multiple features including time, and to view split screens for efficient comparative analysis. The BioNetApp software is freely available under GNU GPL license and can be freely downloaded at the provided link (including the case-study data and user-manual).

### Project management

The BioNetApp software allows intuitive mapping of experimental results into the system by the concept of a project and a panel for managing the project information and samples data. Each project may contain multiple experiments, each with its own sample data. Experimental information is specified as part of the input including fields such as the analytical platform and MS-mode. Samples names are imported automatically when an experiment is imported into an analysis project. Samples features, such as time and group, are specified by the user and automatically saved into the project. This information acts as meta-data during the analysis phase. A single window of BioNetApp is dedicated to displaying data from a single project at a time, with the ability of opening multiple project windows simultaneously.

### Input files format support

Users are able to create a new project and import data into it or to an existing project, with the data being from various platforms, given it is represented in a comma or tab delimited file. A dialog box allows the user to select which columns of the input file represent the samples response data. This data becomes part of the project and is stored to disk for direct access. To prevent errors, BioNetApp only allows columns which consist entirely of numerical data to be selected as expression data columns. Entered sample metadata can also be amended at any time and used as the basis for sample grouping.

The data obtained from the experiments is handled in two different files. The first file is “Normalization.csv” which includes all the molecular expression information for all samples used to conduct the experiment, and also any meta-data information about each molecule including identification information. This file is usually ready for analysis and contains data that has already been aligned and normalized (hence the name) in a previous step using the instrument vendor’s software or in-house developed tools. The Normalization file is the main input to the analysis. Each line of the input consists of a comma separated list of the (customizable) molecule ID, followed by the molecule concentration values in all the samples in the experiment. Each sample concentration is then mapped into the BioNetApp system where scientists can control its participation in the analysis.

The second input file, “project_info.csv”, includes the name and details of the experiment, including the experiment ID, description, analytical platform, MS method and mode, time unit (for time-course data), samples’ IDs used in the experiment, meta data, and any special remarks about the experiment. This file describes features (metadata) about the samples and experiment groups, such as group category, time, radiation, background, gender, age, drug, and race. This versatile metadata allows users to run comparative, correlative, and clustering analysis on the data by choosing the features they are interested in to design the test groups.

BioNetApp allows saving all experiment analysis results to persistent storage. This in turn provides a convenient way of performing experiments and saving the outcomes for later inspection or for use in publications. Scientists could share interesting analysis images including the graphical layout display and their corresponding information.

### Visual data mining

BioNetApp analysis system provides three main functionalities (detailed in the Results section) to explore molecular expression data and aid in the hypothesis generation process: (i) molecular correlation analysis, (ii) distribution analysis with time-course study, and (iii) data clustering, presented across the experiment time points. The correlation analysis provides strong means for understanding the relationship between molecules. It provides different (network graph) layouts for visualizing the molecular concentration and correlation between molecules, using multiple calculation methods such as Pearson product-moment correlation, non-parametric Spearman correlation [22], and non-parametric Kendall Tau-b rank correlation [23]. Through the graphical interface, scientists can visualize useful information by interactively exploring molecules and correlations, and also dynamically select, hide, and filter displayed molecules and correlations. This makes BioNetApp very unique and functionally rich compared to existing tools in terms of molecular expression data.

As for distribution analysis, it enables integration of molecular expression data across samples, groups, and also time points with boxplot display, outlier detection, and data curve fitting. This integration is done based on common attributes found between molecular expression data in the selected time points. And finally, data clustering allows analysis of how a sample concentration changes at different time instances. Since the clustering graph represents the change in concentrations of hundreds of molecules over time, it cannot be easily comprehended visually. Accordingly, those molecules are clustered based on the similarity of changes in their concentrations. This avoids having an overwhelming set of molecules on a graph to visualize the concentration change over time. Instead, the results can be presented as a clustered set of molecules displayed as one line on the graph over different time spans. BioNetApp provides four algorithms for clustering: Self Organizing Maps (SOM), K-Means clustering, K-Medoids, and Farthest First.

### Experimental data

The test case data used here is from metabolite abundance profiles of mouse liver extracted and measured on a linear trap quadrupole—Fourier transform ion cyclotron resonance mass spectrometer (LTQ-FTICR MS) equipped with a chip-based nano-electrospray ionization (nESI) ion source. Exact details of the lab experiments and methods of data pre-processing are described elsewhere[16]. After metabolite peak detection and alignment are done, an aligned metabolite peak table is generated and used as the input to BioNetApp.

The peak table contains information about every metabolite peak, across all the samples used in the experiment, i.e.ID, M/Z, molecule concentration values per sample, and a list of assigned metabolites retrieved via database and MS/MS spectrum matching.

## Results and discussion

BioNetApp provides interactive analysis and graphic visualization of molecular expression data. It provides three main functionalities to explore molecular expression data and aid in the hypothesis generation process: molecular correlation analysis, comparative and data distribution analysis, and data clustering, with time-course study based on molecular concentrations.

**Fig 1:**
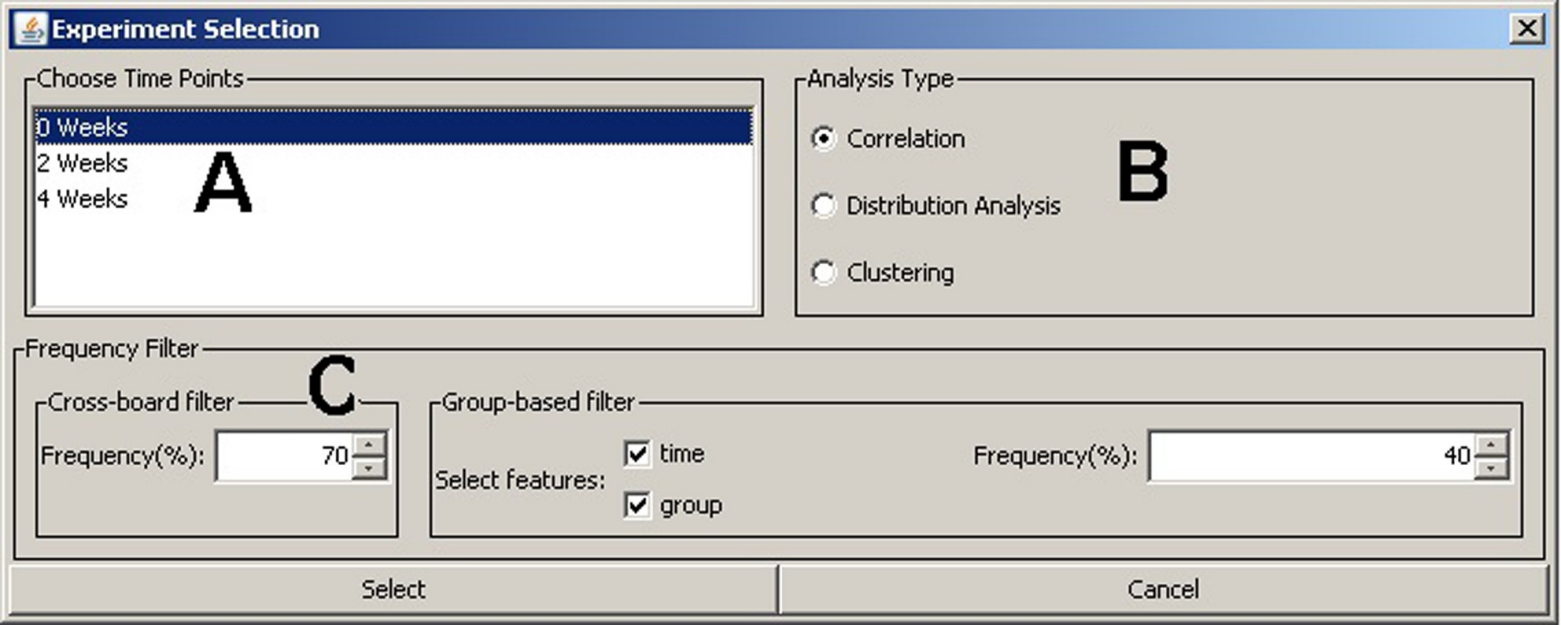
Experiment selection window. (A) Time points available for this experiment (in our case study we chose all three time points). (B) Analysis type to perform. (C) Molecules (peaks) frequency filter across all samples (cross-board filter) and also per group of selected features (group-based filter).

A unique feature of BioNetApp is its ability to analyze data across multiple time points. It provides a selection window for filtering the experiment data based on the meta-features, which includes: timepoints, molecules (peaks) frequency filter, and the analysis type to perform (Fig 1).The “Frequency Filter” uses the meta-features specified as column titles in the project information file and can include features like time, age, radiation, and background. The frequency settings allow to filter data prior to visualization based on data presence frequency in samples and groups, which allows for the omission of molecules that were not detected in a specific percentage of samples, specified by the user. This percentage can be based either on user-specified groups of samples or the overall sample set. The rationale behind this filter is that there are usually many random peaks present in the data that are actually noise. Therefore, we can filter these peaks based on their detection frequency, which equals to the number of samples a peak is detected in, divided by the total number of samples. The filter works on two levels, the first is across all samples in the experiment where at least one group must meet this condition to retain the peak. The second is group-based, where groups that fail to meet the condition will have their molecule concentration values set to zero. The user defines the filter groups by selecting any combination of features from the provided list.

This selection power gives a great flexibility for performing the analysis multiple times with different parameters, while dynamically selecting a portion of the data according to a specific hypothesis, and where the results can be easily observed and compared. There are three types of data analysis as explained in detail next.

### 1) Interactive molecular correlation analysis

**Fig 2:**
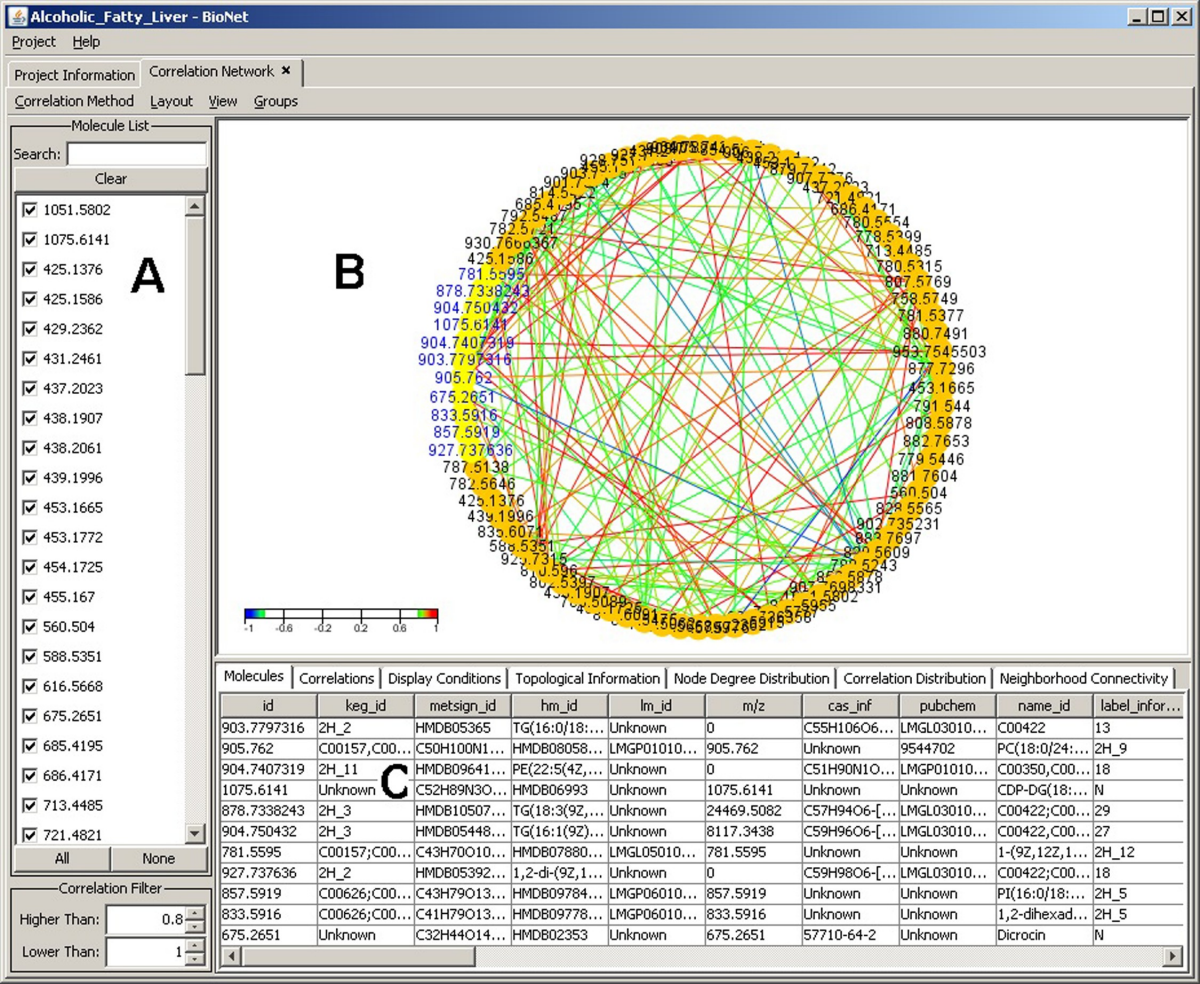
Correlation analysis window with single circle layout. (A) Molecular IDs. (B) Interactive graphic display of molecular correlations in a single circle display while applying the Pearson correlation calculation. (C) Information panel display for rich set of information about the selected (highlighted) molecules in the graph. Color scheme is used to show the correlation directions: red indicates positive correlation while blue indicates negative correlation. The encircled color areas readily demonstrate clusters of strong molecular correlation.

The objective of correlation analysis is to measure how strong molecules are related to one another. After choosing the desired experiment data and the molecules of interest, the correlation visualization module (Fig 2) displays all the molecules available in that experiment with their related information and the correlation between the molecules. This module provides diverse views of the molecules and their relationships by supporting a number of layouts (chosen from the layout menu), such as single or multiple circle layout, random layout, Kamada-Kawai, heat map and Fruchterman-Reingold spring embedding. The single circle layout (Fig 2B)plots interconnected components in ring and star topologies. It is used in applications such as social networking and network management.

Edge coloring between molecules (network nodes) is used to indicate positive and negative correlation values. The color spectrum indicates the correlation value range in terms of colors on the edges. This range enables users to visually spot the differences in correlation in terms of color on the graph instead of inspecting the values of each and every edge.

Random layout is also used to plot molecular relationships in random order. Kamada-Kawai [18] layout is an iterative layout algorithm which starts in a flux state when loaded. This is because it is a force directed layout algorithm that considers the force between two nodes or molecules. This could be represented as rings and springs where the rings denote the molecules and the springs denote the relationships. The main idea of the algorithm is that it attempts to minimize the energy of the system by moving the molecules and changing the forces. The algorithm achieves fast convergence and can be used to layout networks of molecules of different sizes. Fruchterman-Reingold [19] algorithm is often used to obtain a more aesthetically pleasing layout over Kamada-Kawai. It is useful for visualizing large networks and it guarantees that topologically near molecules are placed closer together, whereas far away molecules will be placed further away from each other. Several methods of correlation calculation are supported, such as Pearson, Spearman rank correlation [22], and Kendall Tau-b rank correlation [23].

The correlation visualization includes by default all molecules in an experiment, however, molecules can also be easily selected/unselected from the analysis using the provided molecule list (Fig 2A).For viewing the detailed analysis on a specific list of molecules, the software user can select the molecules of interest directly on the graph(using the mouse) and the selected elements along with all their information will dynamically appear in the molecule information tab (Fig 2C). This enables extended flexibility for the targeted analysis approach. The provided view menu in the interface allows for multiple manipulations of the network graph, like hiding selected or unselected nodes, hiding orphaned nodes, re-displaying hidden nodes, or manipulating the current set of selected nodes. There are also menu items for zooming in or out on the graph, which can also be accomplished using the mouse wheel for ease of use.

Further filtering of what molecules to include in an analysis could be controlled through the main correlation analysis window. Filtering the molecules' list can be performed in two ways. The first is through the molecules' list check boxes: if the user is interested in filtering a specific set of molecules, the search box on top allows for narrowing down this list. The search box supports wild-card matching and regular expressions matching. The second filter is the correlation filter which allows for specifying the range for the correlation values to be visible on the graph. Using a layout such as the provided heat map, it is easy to distinguish any molecule outside the correlation range as it will have a neutral (background) color and its details window will be disabled. The operations mentioned could also be done using the mouse directly to drag nodes, zoom in and out, and select nodes correlated to each other.

The information panel (Fig 2C) contains a detailed set of information about the selected molecules in the graph, including molecule concentrations, current display conditions, and topological information, as well as histograms of various distributions including node degree distribution, correlation distribution, and neighborhood connectivity. The information is organized into a set of tabs that are automatically updated as the user manipulates the graph or changes display conditions through the left side filtering panel and menus. The molecules tab contains a brief description about the molecules currently selected from the graph. The correlation tab contains details about the selected correlations relations in the graph. The topological information panel displays some statistics regarding the currently displayed molecules including the number of correlated molecules and the average number of neighbors. The node degree distribution tab describes the number of neighbors for each molecule in the graph. The correlation distribution tab displays the distribution of correlations between molecules on the graph regardless of being visible or not. The last tab is the neighborhood connectivity where it plots the relationship between the number of neighbors a molecule has and the average degree of each of those neighbors.

**Fig 3:**
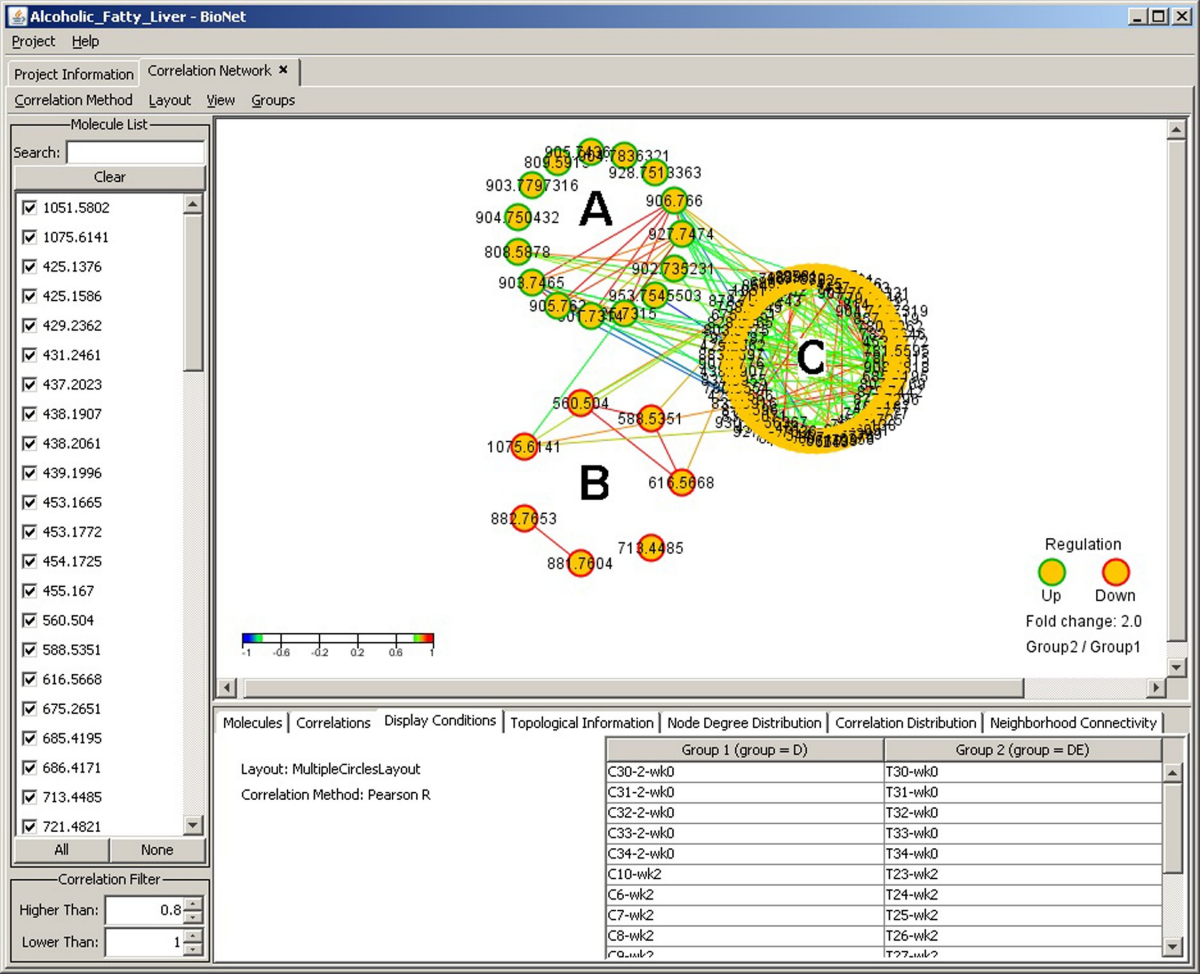
Correlation analysis window with multiple circle layout. Three-circle network graph display illustrating the up-regulated molecules in green borer nodes(A), down-regulated in red border (B), and other (not differentially expressed) molecules with no border (C). The fold change threshold is set at 2, meaning that molecules regulation that don’t meet this threshold are grouped in the third neutral circle (C). The fold change value can be adjusted accordingly.

While single-circle layout displays all molecules in one circle, multiple-circle layout (Fig 3)is used to organize molecules based on the molecular expression data as being up or down regulated between two user-defined groups. In our specific test case where the two groups were defined as the control cohort and the test cohort from the fatty acid liver study, this visualization provides a very powerful distinction and efficient separation of the molecules based on their fold change values across the groups.

**Fig 4:**
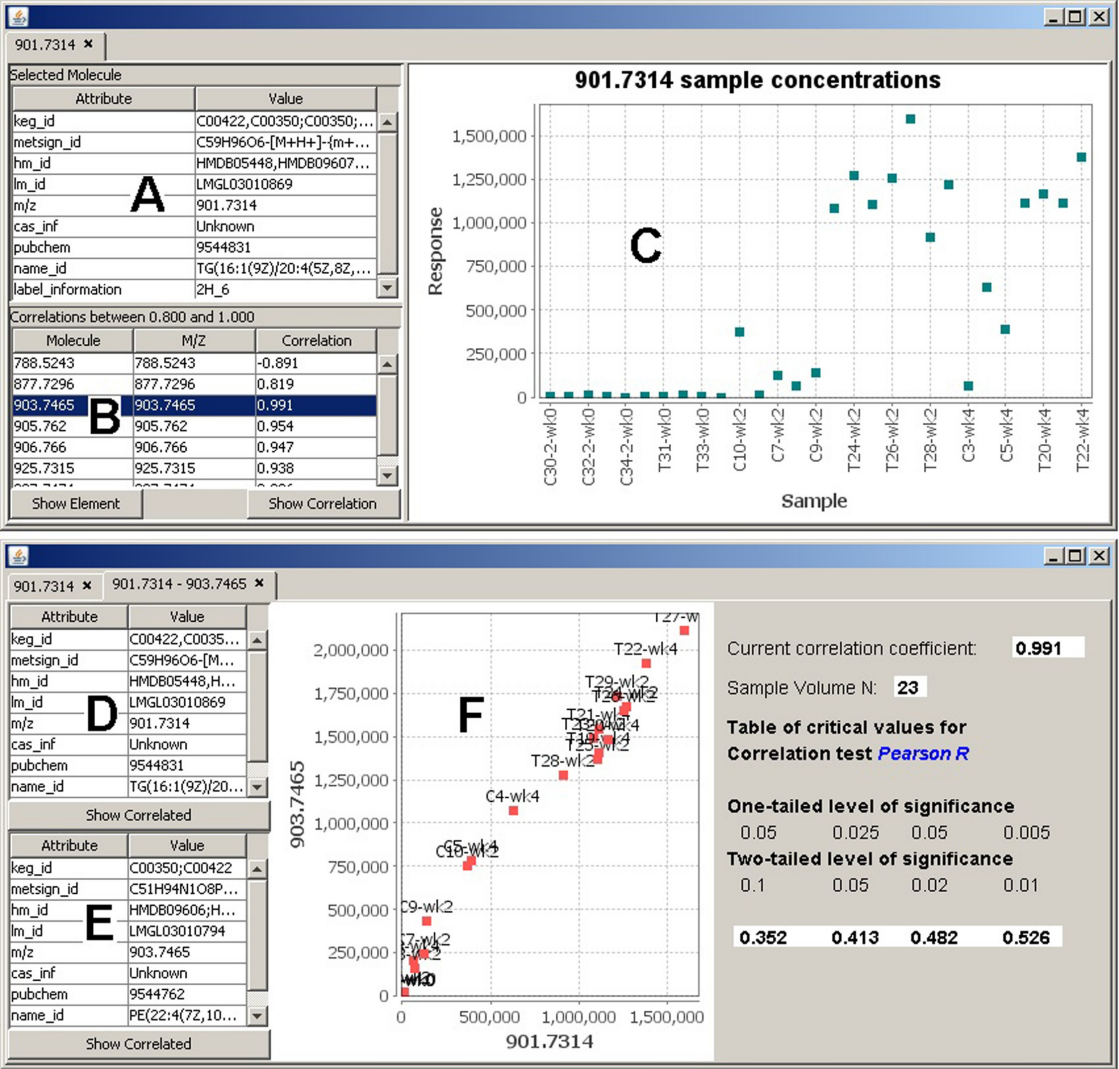
Correlation analysis. (A) Meta information for molecule 901.7314. (B) List of correlated molecules. Further molecule details can be invoked by highlighting a molecule in this list and choosing wither the “Show Element” or “Show Correlation” buttons. (C) Molecular concentration levels across all samples. (D) Concentration details for molecule 901.7314. (E) Concentration details for correlated molecule 903.7465. (F) Correlation graph showing the expression data of the two molecules. Each point represents the expression levels of both molecules (x-axis and y-axis) in the same sample.

Quantitative results in the form of molecule concentrations and correlation information for a selected element can be observed using the sample concentrations plot and the correlation plot, accordingly (Fig 4). In our specific case, we were interested in molecule 901.7314 that is strongly interacting with several other molecules. The concentration visualization instantly provides the attached information for the selected molecule, including its name, label, and identification information (Fig 4A), and a list of the correlated molecules (Fig 4B) with information on their ID, M/Z value, and the correlation value. The correlation graph (Fig 4F) plots the correlations between any two molecules in 2D space. Each point corresponds to the concentration of each sample between the two molecules. It provides important information, such as the used correlation calculation method and the level of significance on one-tailed and two-tailed levels. Both those measures are used in order to describe the statistical significance between the two molecules, assuming a normal distribution. A one-tailed test is used to test the null hypotheses, which means an initial assumption that the significance is based upon. The null hypothesis predicts the direction of the difference. A two-tailed test is used to test the statistical significance of the null hypotheses. The one-tailed probability is half the value of the two-tailed probability.

### 2) Molecular distribution analysis with time-course study

**Fig 5:**
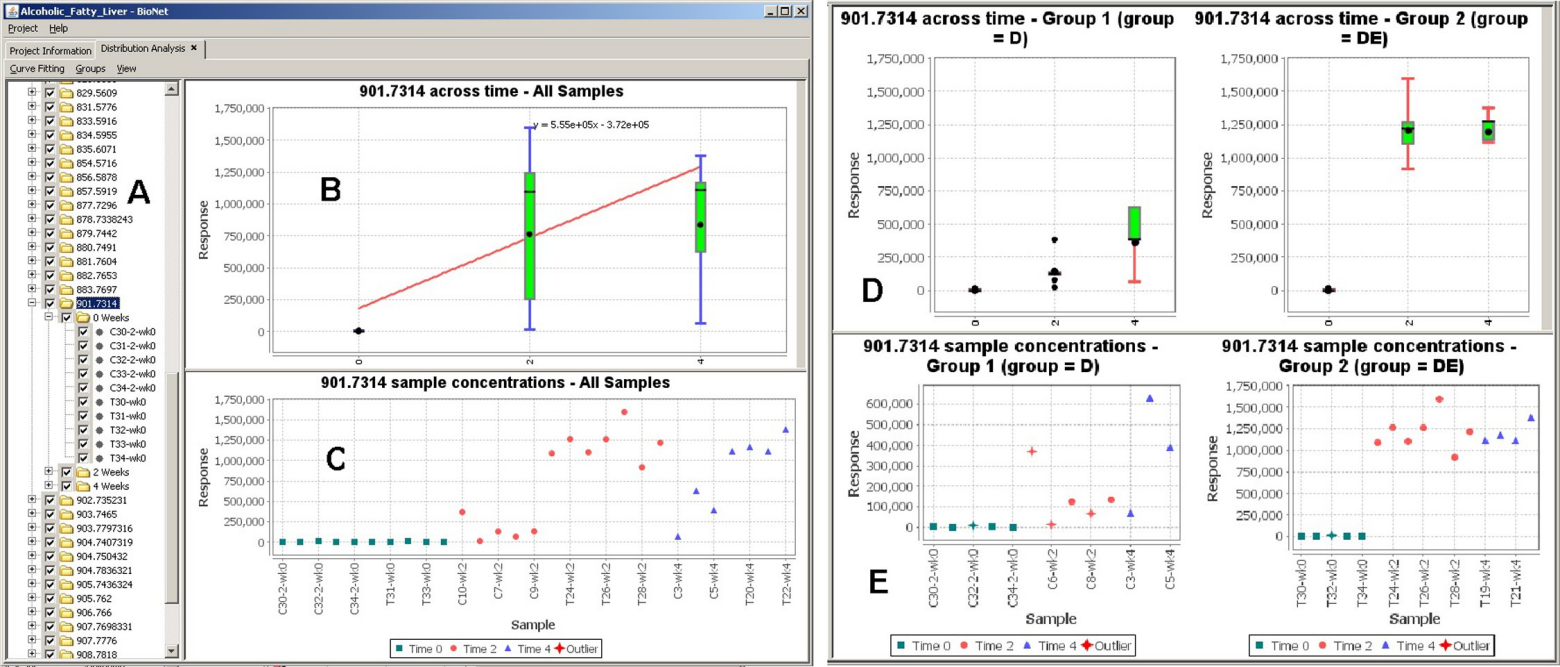
Comparative and distribution analysis. (A) A tree describing the molecules and their corresponding samples and time points. (B) Box plot displaying the concentration of a selected molecule across time points while applying the Robust Linear fitting. (C) Concentration distribution plot of the molecule across all samples. (D) and (E) display the same plots but for two sample groups (control and test) side-by-side for ease of comparison.

Distribution analysis of molecular expression data enables scientists to integrate experimental results into one unified display. This creates a more global view of the data across all experiments and facilitates comparative analysis. This feature allows scientists to be shielded from the merging details and only concentrate on the aggregated information. It plots expression-data distribution across samples, groups, and time points, with boxplot display, outliers detection, and data curve fitting using either Robust Linear or Chi square fitting (Fig 5).

The comparative and distribution analysis information may be observed using a boxplot displaying the concentration of the selected molecule across time points while for example, as in our specific case, applying the Robust Linear fitting (Fig 5B) and a scatter plot of the molecules concentrations across all samples (Fig 5C). This provides a refined method of analyzing how a sample concentration varies for a certain molecule across time points. The interactive interface in BioNetApp allows to select/deselect single molecules, samples, and even time points from the tree view (Fig 5A) to dynamically explore its effect at any given time. The quantitative results of two sample groups (e.g. control and test) can be observed side-by-side for efficient comparison of multiple time points and samples (Fig 5D and 5E).

### 3) Data clustering with time-course study

Data clustering is used to measure how concentrations change for molecules over time and to cluster similar molecules together based on their concentrations. The basic idea is to capture similarities between groups of molecules that share similar concentration trends and patterns and illustrate them across different time spans. The objective is to model the concentrations in order to discover similarities between the molecules represented in clusters.

Clusters generation passes through a set of phases in order to create the final graph. The first phase deals with reformulating the internal molecule representation into one molecule per line on the graph. This means that we represent all the concentrations obtained into one entry per molecule. This allows us to capture the concentration trends for each molecule across samples. The next step is to determine similar molecules by performing the clustering.

Several clustering methods are implemented in BioNetApp including Self Organizing Maps (SOM) and K-Means clustering for identifying clusters of molecules in the data. SOM is a powerful clustering mechanism used for data mining tasks involving high dimensional datasets. It initially populates its clusters by randomly sampling the data based on the initialization condition and then refines the clusters based on its objective function. K-Means clustering on the other hand partitions the observations into k clusters with each observation belonging to the cluster with the nearest mean value. Unlike K-Means, SOM does not require specifying the number of clusters beforehand. Another advantage of SOM is that it provides some information on the similarity between nodes in its map. In order for clustering to capture the similarity between molecules, the input data is modeled one molecule per entry. This configuration allows accommodating of much information and samples as possible for each molecule in order for the clustering algorithm to discriminate between molecules.

**Fig 6:**
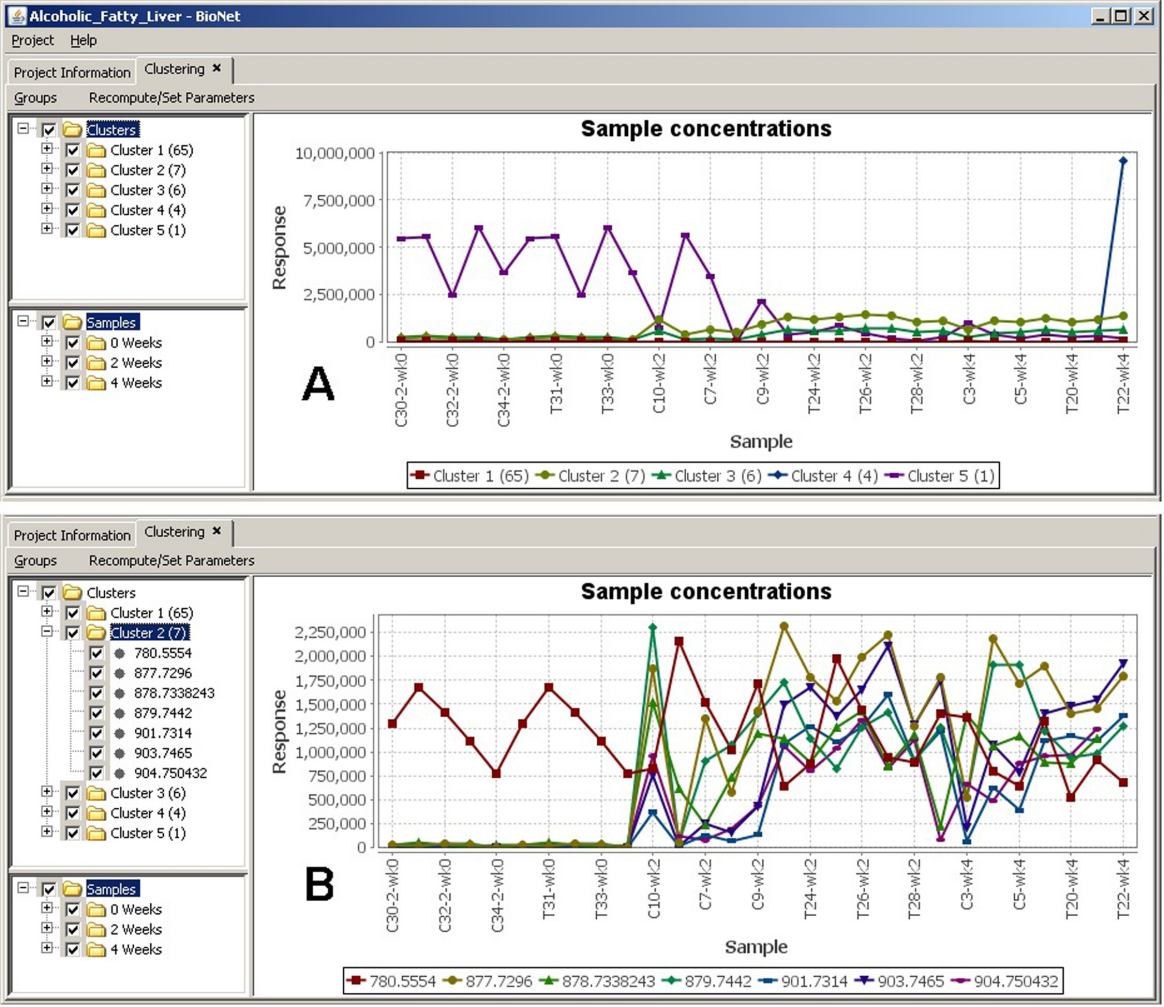
Clustering. (A) Clustering averages across all samples, (B) Cluster 2 molecules concentration details across all samples.

The output of clustering is a numerical representation for each molecule indicating its similar cluster. The generated clusters are further used in order to plot the clustering graph (Fig 6). This is done by taking the average concentration for molecules in the same cluster per sample. Other measures could be used instead of average including the mode, maximum, and minimum concentrations. In our specific test case, clustering was generated to study the differences of the metabolite expression profiles acquired under different physiological conditions, with temporal analysis focused on the trajectory correlation of each metabolite between the sample cohorts. Five clusters are detected in the data (Fig 6A), where we were able to quickly identify interesting patterns. In this example we are interested in molecules that become up-regulated across groups. Cluster 2 shows the highest expression values while being up-regulated across time points (samples are sorted on x-axis by time points first, then alphabetically).This analysis can be taken further by exploring the molecules grouped in this cluster (Fig 6B). This detailed view offers a quick way to inspect the molecules expression values across samples, in both control and test groups, and to identify interesting ones. Similar to the functionalities in the distribution plots, two groups can also be set to display two side-by-side graphs representing each group, to easily compare differences in data patterns across groups and time points. This functionality allowed us great efficiency in studying the data and discovering interesting patters and behaviors.

## Conclusions

The objective of our work is to enhance the experience of users when performing data analysis with a large amount of data, including time-points, by providing powerful visualization capabilities that are integrated with data mining and statistical techniques. The BioNetApp software takes data from high volume molecular expression experiments as its input and enables interactive visual data mining of molecular correlations, comparative, and clustering analysis with time-course study. It provides a project management GUI interface that enables users to easily manage different projects, import experiment data, and manipulate the meta-data associated with the project and experiment samples.

The correlation functionality supports several visualization features and is presented with circular and heatmap layouts. The software provides a common framework, allowing presentation of molecular correlations from multiple omics experiments in a single environment. The user is capable of intuitively and interactively manipulating the data during visualization, such as restricting the viewed items based on correlation strength, selecting and hiding nodes, highlighting neighbors, and selecting molecular expression regulation sub-networks. An information panel presents molecule/correlation details, topological information, and graphs for node-degree distribution, correlation distribution, and neighborhood connectivity. Search can also be performed to filter out molecules from selection.

BioNetApp also provides the capability for comparative analysis of molecular expression data through analyzing expression-data distribution across samples, groups, and time points with a boxplot display, outlier detection, and data curve fitting. It also provides data clustering features based on molecular concentrations using Self Organizing Maps (SOM), K-Means, K-Medoids, and Farthest First algorithms, with time-course projections. All produced graphic presentations can be exported as image files for printing or use in publication, as well as the molecular correlation information that can be exported in a simple flat text format.

As a data mining tool for molecular expression studies, the BioNetApp software has been successfully used in our case to indicate elemental level correlations and to investigate the metabolite abundance changes in alcohol induced fatty liver.

## Supporting information

S1 File BioNetApp.zip. The BioNetApp software including the case-study data and user-manual.
(ZIP)
