# Supplementary material for: BioNetApp: An interactive visual data analysis platform for molecular expressions
Source: PLoS One. 2019 Feb 22;14(2):e0211277. doi: 10.1371/journal.pone.0211277 (PMC6386483; doi:10.1371/journal.pone.0211277)
Supplement: S1 File — The BioNetApp software including the case-study data and user-manual. (ZIP) [file pone.0211277.s001.zip › BioNetApp_Manual_ReadMeFirst.pdf]

BioNetAppUser Manual

About BioNetApp

BioNetApp is an interactive visual data mining application software for analyzing intermolecular correlations using various statistical methods. BioNetApp can perform interactive comparative, correlative, and time-course analysis of molecular expression data.

The BioNetApp software is freely available under GNU GPL license, and is included in the downloadable folder under the name “BioNet.jar”.

This work was partly funded by NIH 1RO1GM087735, Purdue University Cyber Center, and Purdue Bindley Bioscience Center.

The BioNetApp Development Team

Table of contents

About BioNetApp

References

1.How to open an existing project

2. Performing analysis

3. How to create a new project

1. How to open an existing project

First make sure that you have the Java runtime environment installed (https://www.java.com/). Then you can run the BioNetApp application by double clicking the executable Java file with extension ".jar", for example "BioNet.jar".

If your experiment data is saved in a separate file, go to "Project" at the top left menu and select "Open Project" (see figures 1a and 1b below). When the "Open" window is displayed, choose the file type as "project" instead of "All Files" to filter the files.

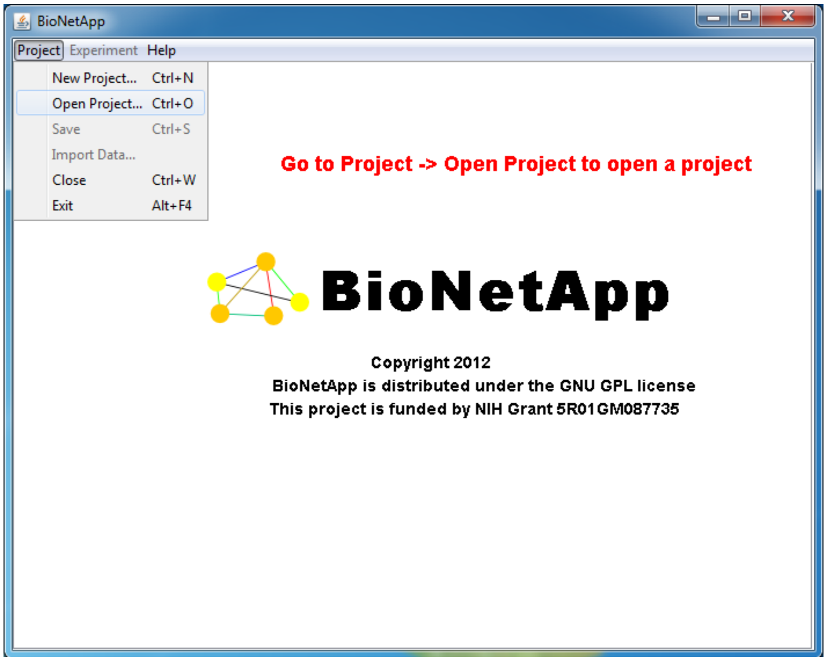

Figure 1a: Open Project.

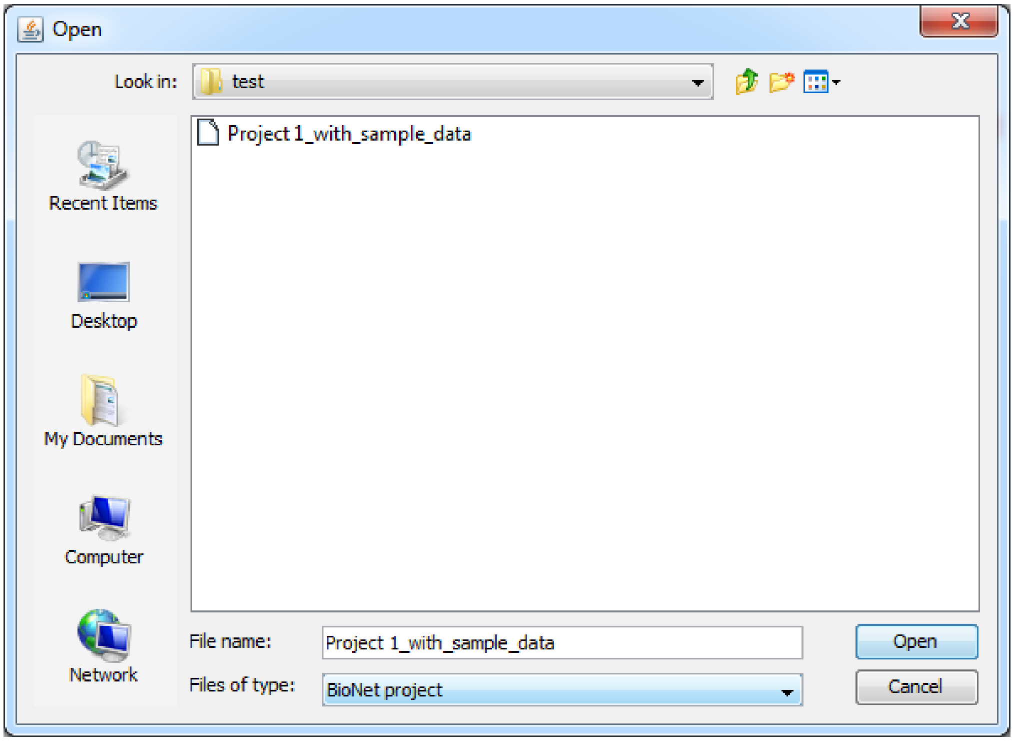

Figure 1b: Open Project- choose file type**.**

The "Project Information" page will be displayed showing names of all samples and their corresponding group and time points (see Figure 1c). The user can modify the displayed project and samples information as needed.

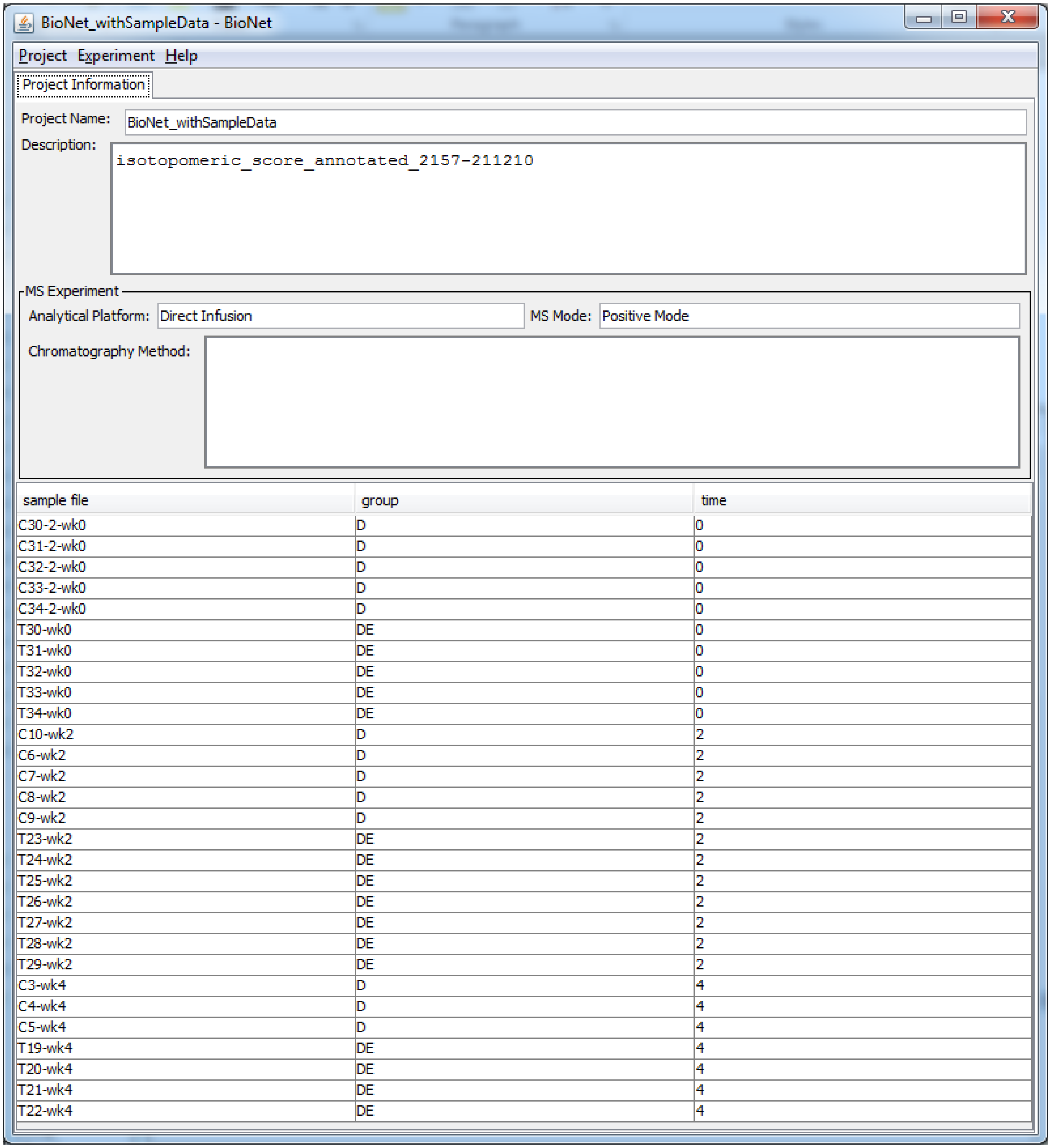

Figure 1c: Open Project– Information page**.**

2. Performing analysis

Once a dataset is selected, the user can go to "**Experiment**" from the top left menu and select the parameters for his experiments as shown in Figure 2. Top left block shows the time points available for the experiment. It is important that the user choose all time points they are interested in analyzing, as only those chosen will be included and available as criteria for creating groups of samples later on.The right block is for selecting the type of analysis to perform as correlation, distribution, and clustering analysis. The bottom block controls molecules (peaks) frequency filter across all samples and also per group.

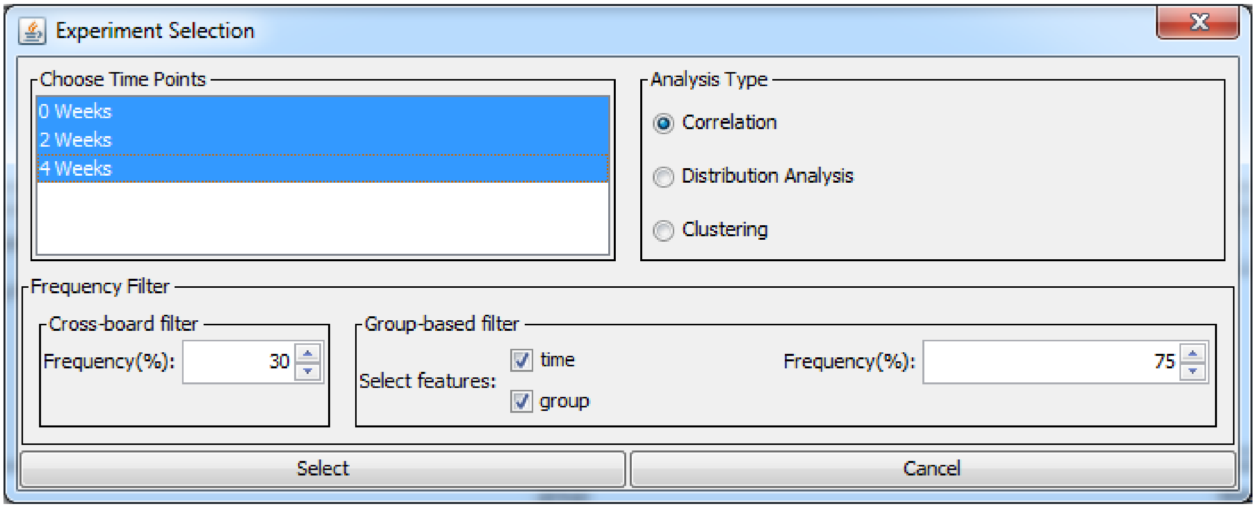

Figure 2: Experiment Selection Window.

After setting the experiment analysis parametersthe user can choose, for example, Correlation Analysis method as shown in Figure 3. The objective of correlation analysis is to measure how strong molecules are related together. After choosing the desired experiment data and the molecules of interest, the correlation visualization module will be displayed. For example, Figure 3 shows Correlation Analysis window with single circle layout alongwith the related information for all the molecules available in that experiment.

There is also a multiple circleslayout option for representing the upregulated, downregulated, and neutral expression data, based on the correlation filter, which can be modified (bottom left of the interface screen). In order to use this feature, the user must first separate the samples into two groups based on the criteria of choice or a combination of them. This is done by clicking the ‘Groups’ tab and selecting ‘Choose Sample Groups’. For example, the user can choose to view the correlations between samples in the control group versus experimental group, or based on all the samples but at different time points, or even based on any combination of the criteria provided with the samples.

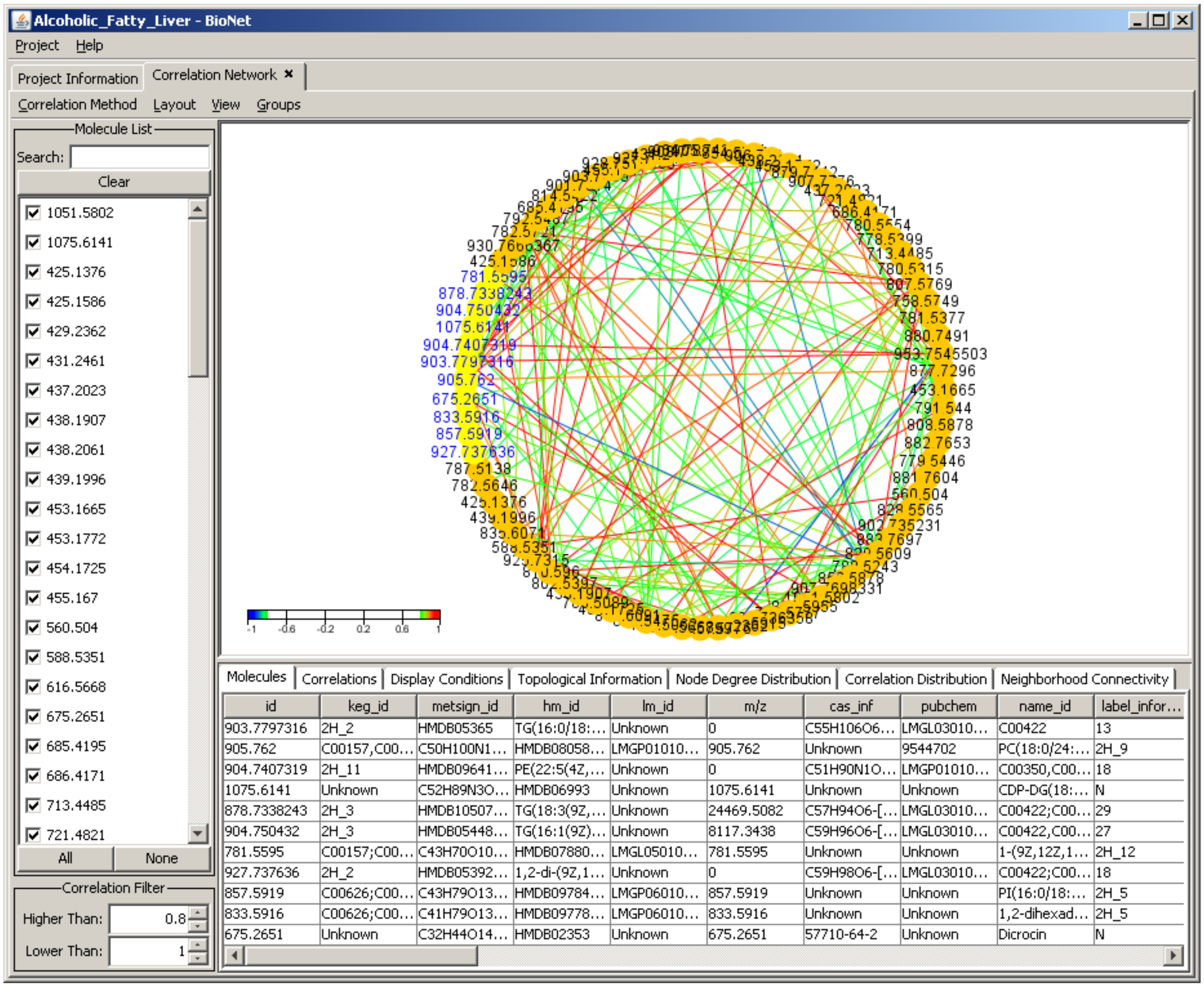

Figure 3. Correlation Analysis Window with Single Circle Layout.

Further analysis can be pursued by the BioNetAppsoftware by highlighting any molecule in the display panel, then either clicking the “Show Element” button toinvokethe concentration detail window of the highlighted molecule, or clicking the “Show Correlation” button to invoke a correlation window, as shown in Figures 4a and 4b, respectively.

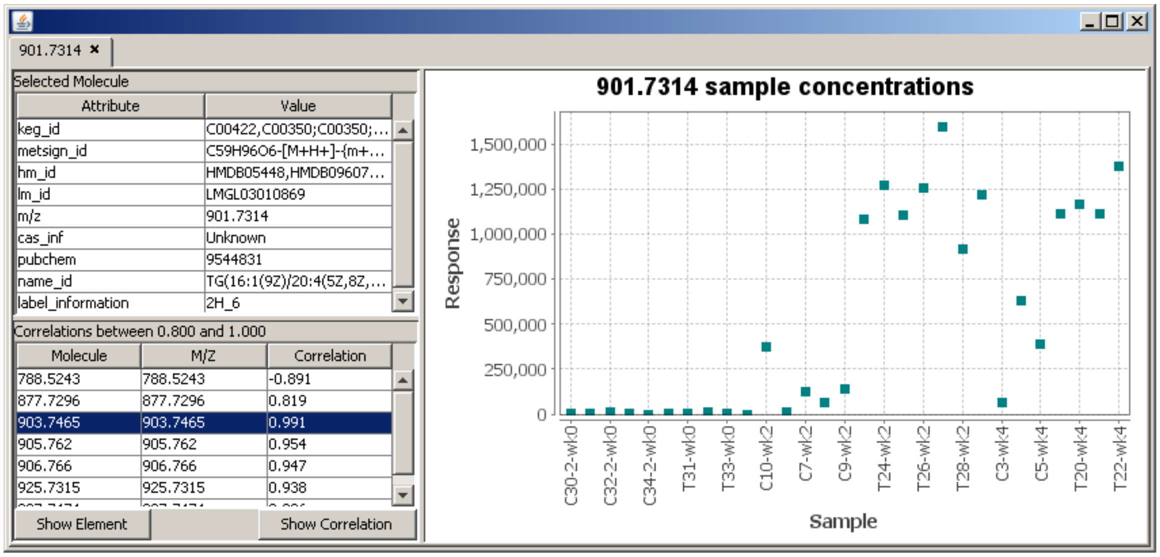

a)

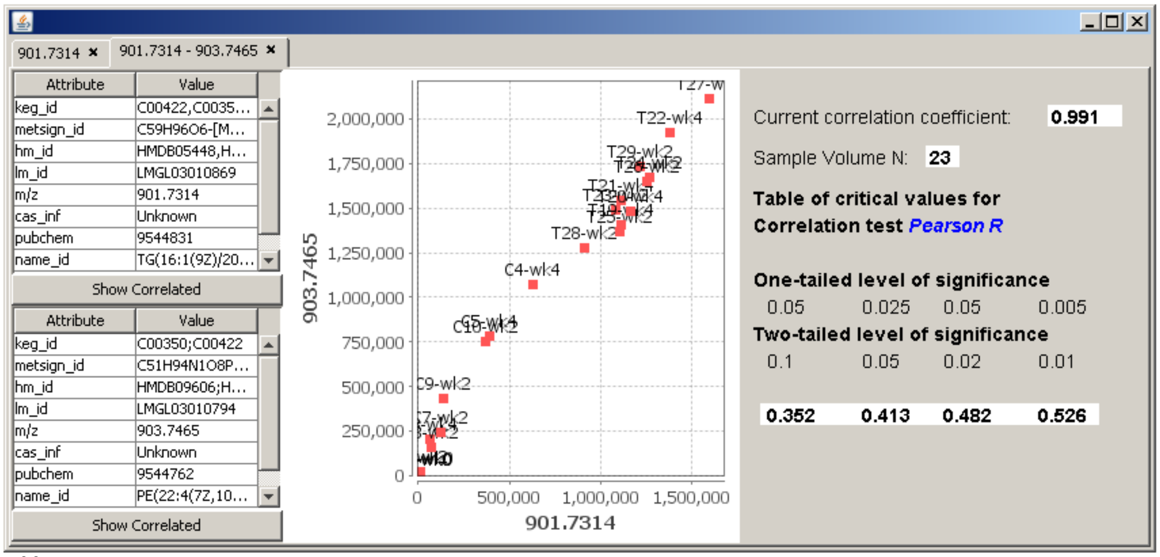

b)

Figure 4: Correlation Analysis: a) Concentration details for a single molecule, **b)** Correlation analysis between two molecules**.**

The user can select another type of analysis for the data, such as Distribution Analysis. Distribution analysis of molecular expression data plots expression-data distribution across samples, groups, and time points, with boxplot display, outliers' detection, and data curve fitting using either Robust Linear or Chi square fitting. It enables scientists to integrate experimental results into one unified display. This creates a more global view of the data across all experiments and facilitates comparative analysis. This feature allows scientists to be shielded from the merging details and only concentrate on the aggregated information.

Figure 5 shows, on the left sidebar, a tree describing the molecules included in the experiment data and their corresponding samples and time points. The user can select/deselect the molecules and their samples or time points accordingly from the tree view. Users can explore the relationships between each selected molecule at any time point. It is also possible to control the viewed items on the sample level.

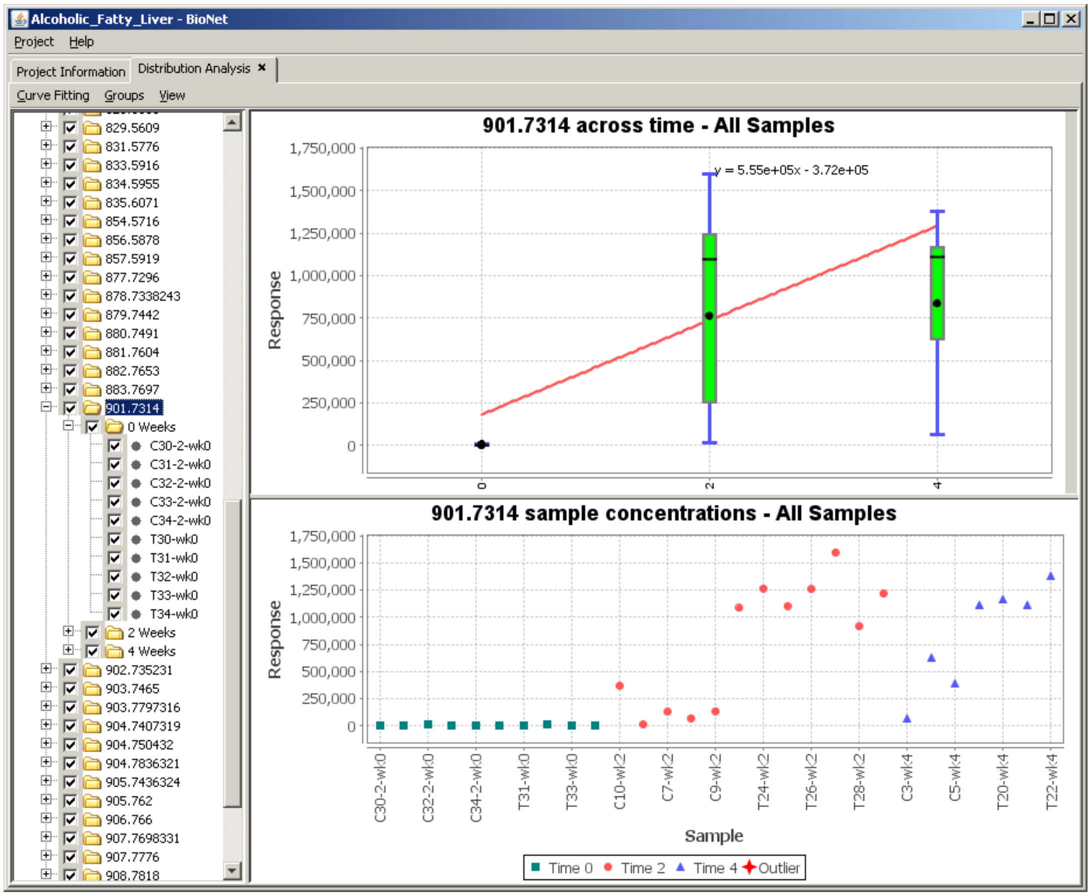

Figure 5: Comparative and Distribution Analysis.

The third analysis type, Data Clustering,can be used to measure how concentrations of molecules change over time, and to cluster similar molecules together based on their concentrations (see Figure 6). The basic idea is to capture similarities between groups of molecules that share similar concentration trends and patterns and illustrate them across different time spans.

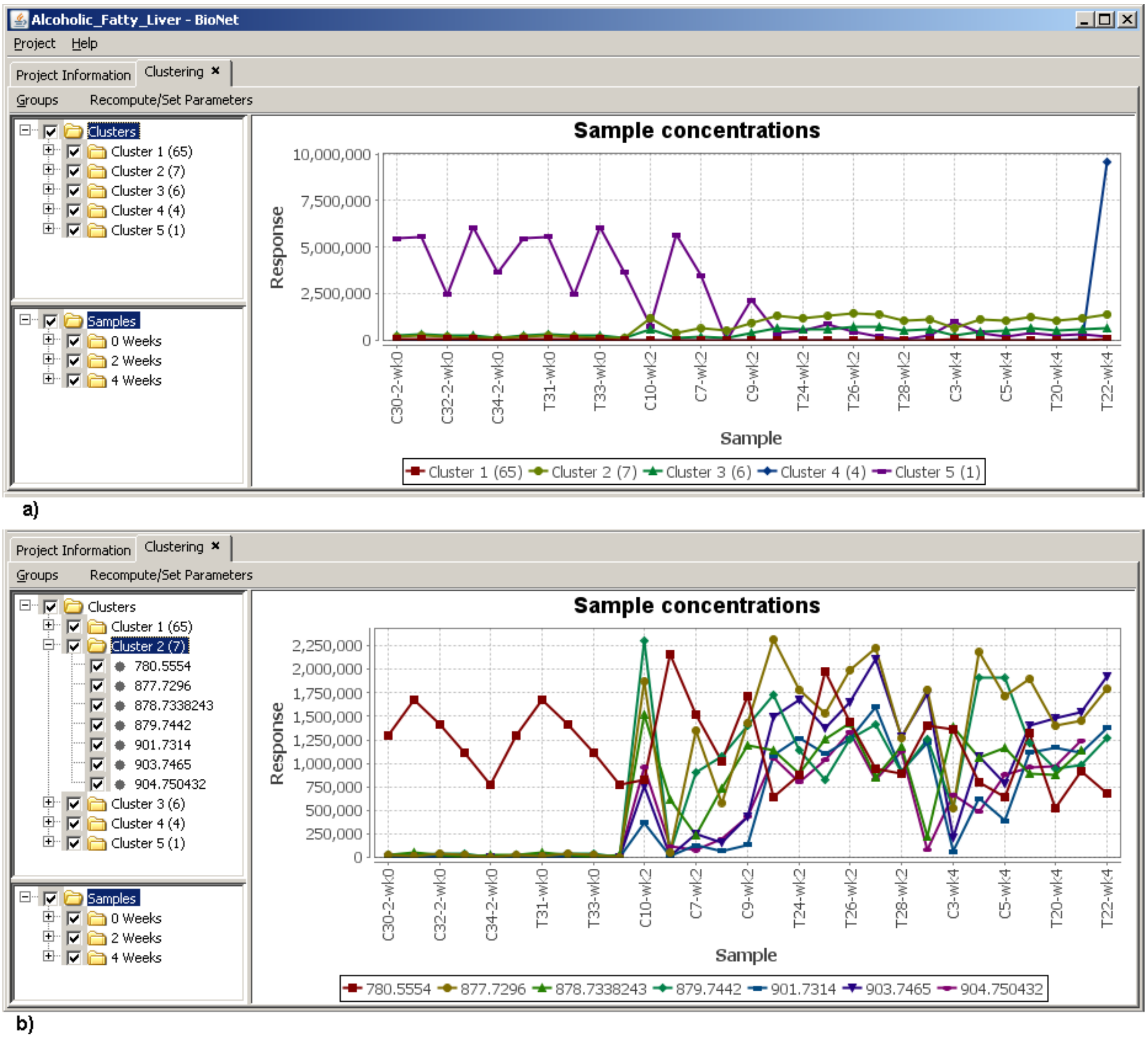

Figure6. Clustering: a) Clustering averages accross all samples, **b)** "Cluster 2" (only) molecules concentration details across all samples.

3. How to create a new project:

To create a new projectthe use needs to go to “Project” at the top left menu and select “New Project” (seeFigure 7 below). When the “Save” window is displayed, the user can choose a name for the projectfolder and the location where it will be stored.

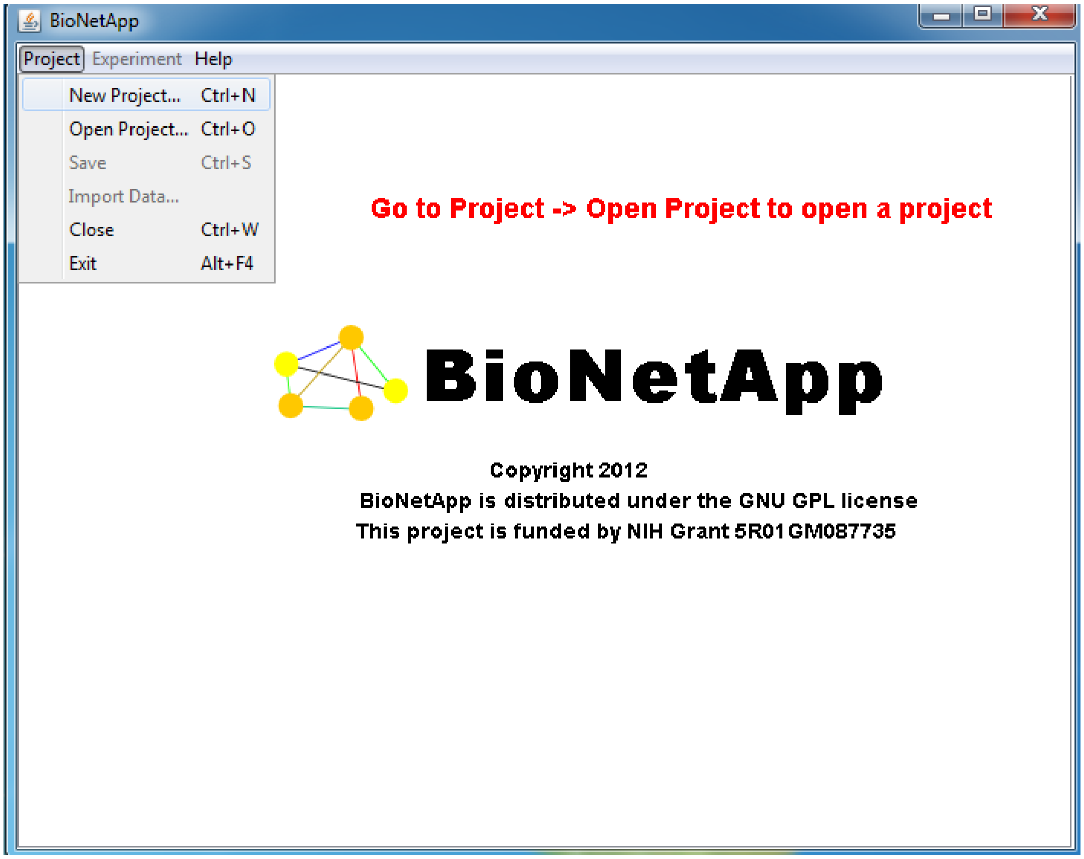

Figure 7: New Project.

Once the “Save” button is pressed, the project information screen will be displayed as shown in Figure 8. The user may want to fill in related information for the project, then click save again. On the technical side, a new folder with the project name will be created which includes a stylesheet project information file called “project_info” and a Normalization folder that will later host all experiment files that are imported to the project.

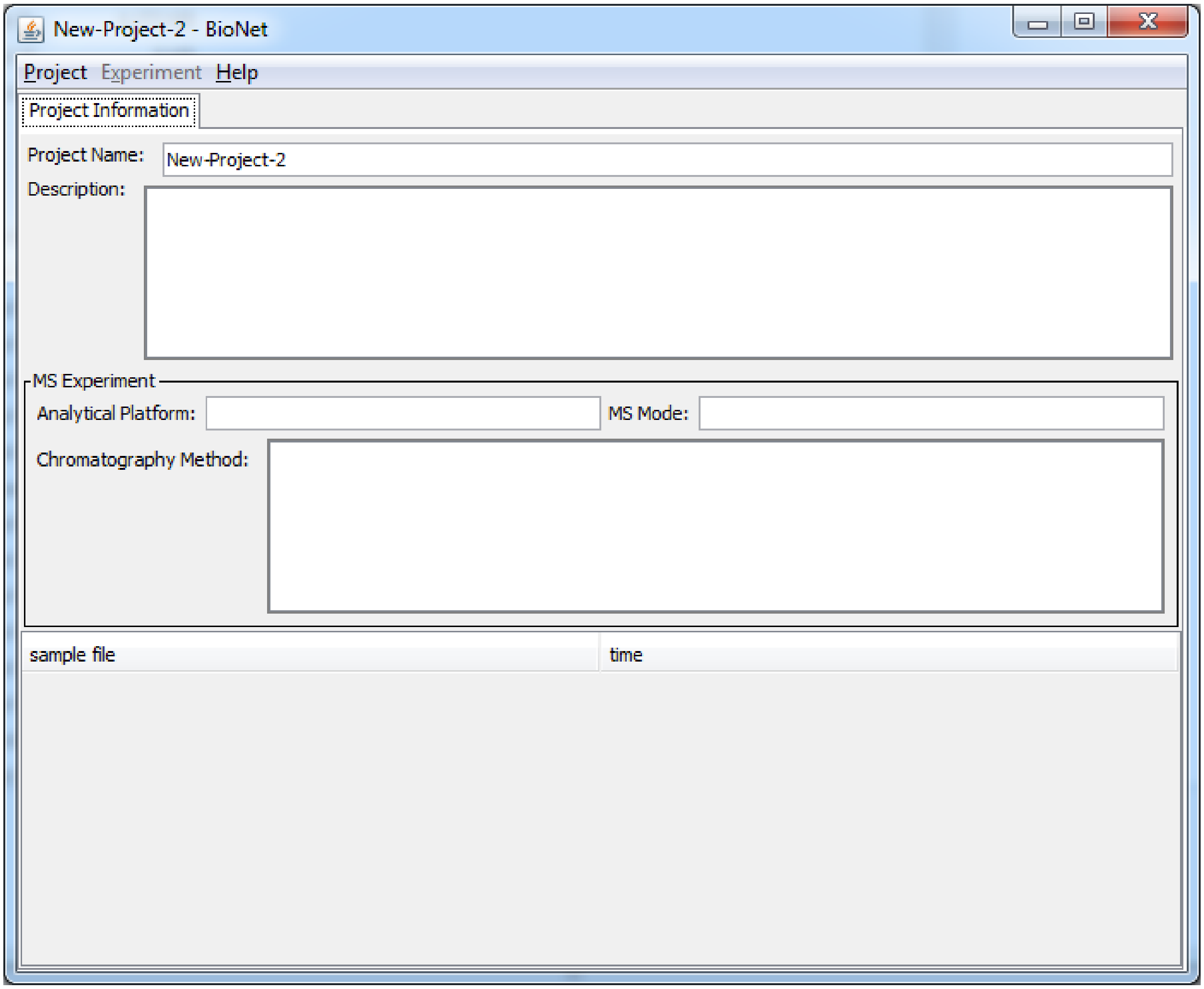

Figure 8: New Project Information Page.

To import data to the project, under the “Project” menu item, choose “Import Data” and a window will pop up (shown in Figure 9a) where the use can choose the desired input file with the samples data.

Another window will pop up displaying all the columns in the file. At this point the user must specify an “Experiment Name” and also deselect any column that do not represent samples data as valid molecular expression values as shown in Figure 9b.

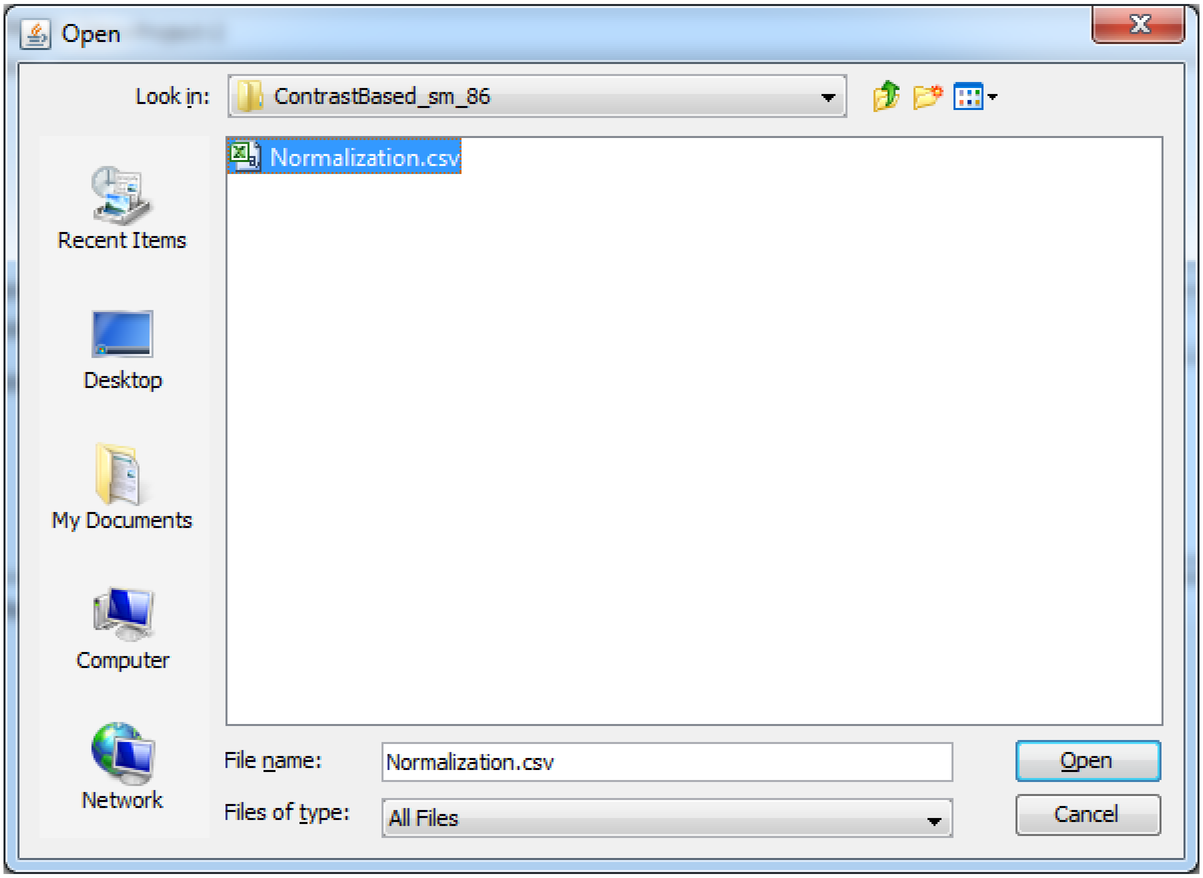

Figure 9a: Importing Samples Data.

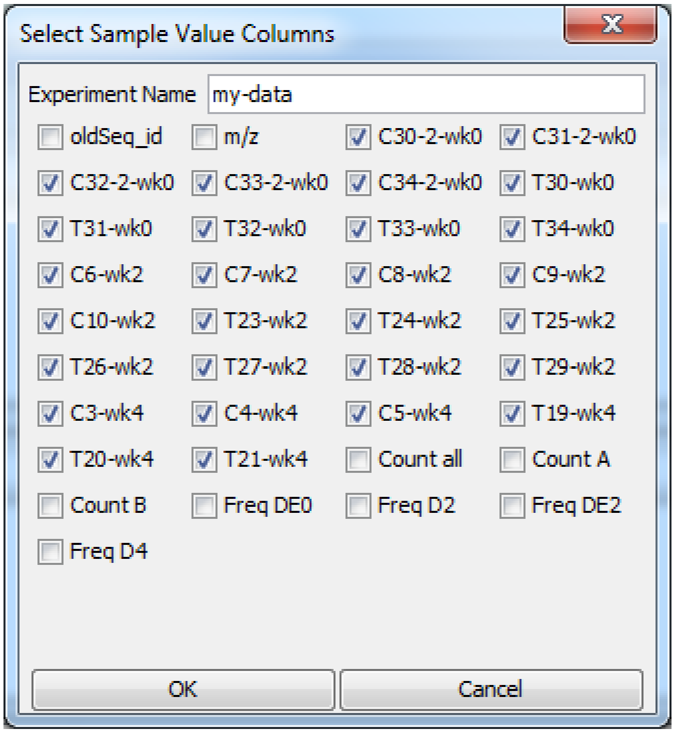

Figure 9b: Deselecting columns that are not samples.

Finally, the updated “Project Information” page will be displayed showing names of all imported sample and a default single time point set as “0” for all samples. If the user would like to specify different time points for the samples (or any other criteria), he/she can do so on the “Project Information” window by changing the values under the “Time” column in the lower table. Users can also add new categories for grouping samples later, like “group”, and specify desired values in front of each sample to distinguish it as, for example, disease versus control, similar to Figure 1c. The user can add and remove columns as needed. This information for distinguishing samples is essential later for performing comparative analysis. For example, when performing Correlation Network analysis, the user can group samples as control versus disease and compare the molecular expression changes as up-regulated versus down-regulated between the two groups of samples.
